# G3BPs in Plant Stress

**DOI:** 10.3389/fpls.2021.680710

**Published:** 2021-06-10

**Authors:** Aala A. Abulfaraj, Heribert Hirt, Naganand Rayapuram

**Affiliations:** ^1^Department of Biological Sciences, Science and Arts College, King Abdulaziz University, Jeddah, Saudi Arabia; ^2^King Abdullah University of Science and Technology (KAUST), Thuwal, Saudi Arabia; ^3^Max Perutz Laboratories, University of Vienna, Vienna, Austria

**Keywords:** RNA metabolism, G3BPs, stress granules, P-bodies, RNA regulation, post-transcriptional regulation, RNA-binding proteins, translational control

## Abstract

The sessile nature of plants enforces highly adaptable strategies to adapt to different environmental stresses. Plants respond to these stresses by a massive reprogramming of mRNA metabolism. Balancing of mRNA fates, including translation, sequestration, and decay is essential for plants to not only coordinate growth and development but also to combat biotic and abiotic environmental stresses. RNA stress granules (SGs) and processing bodies (P bodies) synchronize mRNA metabolism for optimum functioning of an organism. SGs are evolutionarily conserved cytoplasmic localized RNA-protein storage sites that are formed in response to adverse conditions, harboring mostly but not always translationally inactive mRNAs. SGs disassemble and release mRNAs into a translationally active form upon stress relief. RasGAP SH3 domain binding proteins (G3BPs or Rasputins) are “scaffolds” for the assembly and stability of SGs, which coordinate receptor mediated signal transduction with RNA metabolism. The role of G3BPs in the formation of SGs is well established in mammals, but G3BPs in plants are poorly characterized. In this review, we discuss recent findings of the dynamics and functions of plant G3BPs in response to environmental stresses and speculate on possible mechanisms such as transcription and post-translational modifications that might regulate the function of this important family of proteins.

## Ras GTPase-Activating Protein-Binding Proteins (G3BPs)

Ras-GTPase-activating protein (SH3 domain)-binding proteins get their name from the first identified G3BP1 protein that was shown to bind Ras-GTPase activating protein (RasGAP) (Parker et al., 1996). The Ras family of GTPases, which are key signal transducers, activate serine/threonine kinases such as Raf to initiate downstream signaling. Hydrolysis of the Ras-bound GTP molecule to GDP by RasGAPs, inactivates Ras thereby suppressing signaling. G3BPs are also known as Rasputins (RIN) in Drosophila and mosquitoes of the genus *Aedes* and *Anopheles*, which are conserved throughout eukaryotic evolution as being members of the family of heterogeneous nuclear RNA-binding proteins and components of the Ras signal transduction pathway (Fros et al., 2015; Krapp et al., 2017; Alam and Kennedy, 2019; Laver et al., 2020). There are two G3BP genes in mammals namely, G3BP1 and G3BP2, whereas there is a single gene, Rasputin (RIN) in Drosophila. Overexpression of G3BPs in human cells and Drosophila S2 cells induces the formation of stress granules (SGs) even in the absence of stress (Tourrière et al., 2003). It was shown that human cells lacking both G3BP1 and G3BP2 were unable to form SGs in response to the phosphorylation of eukaryotic initiation factor eIF2α or the inhibition of eukaryotic initiation factor eIF4A. However, they were found to be a SG-competent after challenging with heat or osmotic stress. Furthermore, human G3BP is able to interact with 40S ribosomal subunits through its arginine-glycine (RGG) rich motif essential for G3BP-mediated formation of SG. Several viral and host proteins that contain FGDF motifs bind to G3BP and alter its physical state and also block the formation of SGs (Kedersha et al., 2016). The host protein G3BP functions as an important proviral factor (Scholte et al., 2015; Kim et al., 2016; Schulte et al., 2016; Götte et al., 2019). The C-terminal domain of the viral non-structural protein 3 (nsP3) of Semliki Forest virus (SFV) forms a complex with mammalian G3BP and segregates into viral RNA replication complexes leading to the inhibition of the formation of SGs. The binding domain of nsP3 to HsG3BP shows two tandem “FGDF” repeat motifs adjacent to the C-terminus of the viral proteins (Krapp et al., 2017). When either of the phenylalanine residues is mutated, they lose the ability to bind to G3BP. The binding of G3BP to FGDF motifs is conserved among Old World alphaviruses (Panas et al., 2015) and the interaction with G3BPs is essential for the replication of many of these viruses (Götte et al., 2020).

## Structural Motifs of G3BPs

Structurally, G3BPs are composed of four distinct domains that are conserved among all eukaryotic G3BP family members—a nuclear transport factor 2 (NTF2) like domain, a central acidic and proline-rich region (PxxP), an RNA recognition motif (RRM) and an arginine and glycine rich box (RGG box). The NTF2 domain is a small homodimeric protein domain, which is involved in RanGTP-dependent nuclear import of proteins through the nuclear pore complex (Suyama et al., 2000; Pamonsinlapatham et al., 2009; Alam and Kennedy, 2019; Reuper et al., 2021). NTF2 was first identified as a factor that stimulates efficient import of proteins into the nucleus and mutants in NTF2 disrupt the NTF2-mediated import of nuclear proteins. The interaction between NTF2 and Ran is crucial for efficient nuclear import of proteins, whereby the inactive GDP bound cytoplasmic form of the small RanGTPase switches to the active GTP bound form in the nucleus. Thus, cycling of the nucleotide-bound state of Ran forms a gradient that stimulates nucleocytoplasmic transport and acts as a cellular marker in distinguishing between the nuclear and cytoplasmic compartments of the cell in the transport machinery. The NTF2 domain is, however, not only important for nuclear localization, but also essential for auto-aggregation, which facilitates the recruitment of the protein to SGs (Quimby et al., 2000; Suyama et al., 2000; Tourrière et al., 2003; Vognsen et al., 2013; Alam and Kennedy, 2019; Reuper et al., 2021). Moreover, NTF2 has been shown to have a role in protein-protein interactions (Tourrière et al., 2003; Alam and Kennedy, 2019). The region of highest variability among G3BPs is the central region, which comprises a varying number of proline-rich (PxxP) motifs and an acid-rich domain. PxxP is coupled with protein interactions and is the minimal consensus target site for SH3 domain binding, which is a small protein interaction module that is essential for signal transducers. The variability in the proline-rich domain among G3BPs suggests that these proteins have different interacting signaling partners (Ren et al., 1993; Alam and Kennedy, 2019). The acid-rich motif seems to be unstructured and it shows similarly with other motifs in some transcriptional factors that have a role in protein-protein interactions (Alam and Kennedy, 2019). G3BP’s C-terminal canonical RNA Recognition Motif (RRM) is involved in RNA binding. RRMs are structural domains identified by two short, loosely conserved motifs, octapeptide RNP1 and hexapeptide RNP2, which interact with RNA via beta sheets whereby the structural integrity is provided by alpha helices. The RRM binds to other proteins which in turn affects the specificity of G3BP for RNA interactions (Kennedy et al., 2002; Irvine et al., 2004; Cléry et al., 2008; Alam and Kennedy, 2019; Reuper et al., 2021). The C-termini of G3BPs also contain an RGG (arginine-glycine rich) domain that is often found in RNA-binding proteins to facilitate RNA binding and influence their nuclear translocation and facilitate post transcriptional modifications (Nichols et al., 2000; Darnell et al., 2001; Alam and Kennedy, 2019). The acidic region, the prolines in the PxxP and the RGG domain are predicted to be intrinsically disordered regions (IDR). These IDRs are necessary for maintaining the saturation concentration of G3BP and the destabilization of the interactions amongst these regions results in the relaxation of G3BP thereby promoting intermolecular protein-RNA and/or protein-protein interactions that drive condensation to form SGs (Guillén-Boixet et al., 2020).

## Molecular Function of G3BPs

**FIGURE 1:**
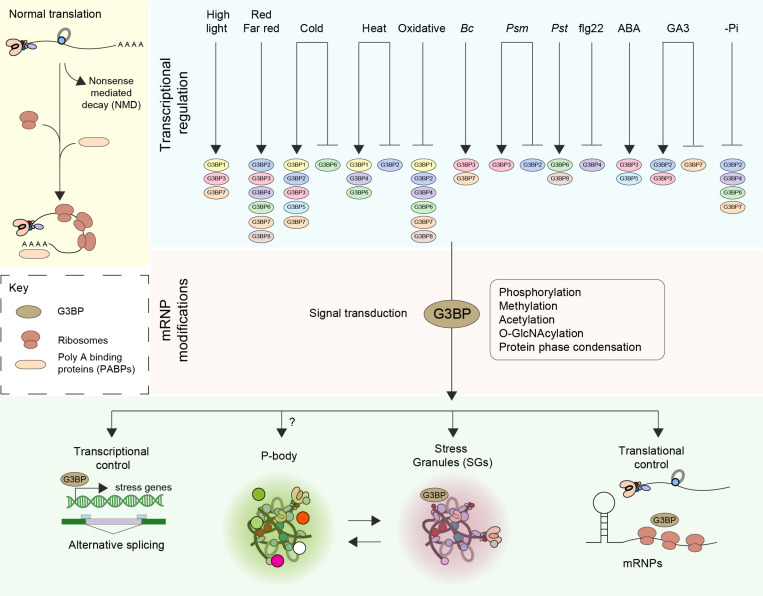
Function and regulation of G3BPs. The fate of mRNA is delicately balanced by means of an equilibrium between translation, trafficking, storage and decay (Nonsense mediated decay–NMD). Perception of various external stress stimuli such as abiotic (light, cold, heat, and oxidative), biotic [*Botrytis cinerea* (*Bc*), *Pseudomonas syringae maculicola* (*Psm*), *Pseudomonas syringae* pv. *Tomato DC3000* (*Pst*)] stresses, elicitors [the flagellin peptide (flg22)], hormones (ABA, GA3) and nutrient starvation (-Pi) results in transcriptional induction or repression of G3BPs. A log2 Fold change > ± 1 was considered. Stresses induce the disassembly of polysomes, thereby freeing mRNAs and mRNPs. The subsequent steps in the signal transduction pathway involve posttranslational modification such as phosphorylation, methylation, acetylation and O-GlcNAcylation of mRNPs. An increase in the pool of translationally inactive mRNA favors the assembly of SG orchestrated by G3BPs. The G3BPs then exert transcriptional control by altering gene expression and/or alternative splicing or by inducing the formation of stress granules (SGs) thereby controlling translation. SGs disassemble when the organism adapts to stress or when the stress is relieved, facilitating the restoration of normal translation.

G3BPs play a central role in the regulation of mRNA including translational control and stability to regulate cellular homeostasis, RNA metabolism, and gene expression at the posttranscriptional level (Figure 1). Recently, however, the importance of the G3BP family has become more apparent in other cellular activities. Human G3BPs were reported to act as multifunctional proteins that interact with other cellular proteins at different developmental stages in different cell types including neurological disease, tumorigenesis and in host antiviral defense (Martin et al., 2013; Zhang et al., 2015; Anisimov et al., 2019; Reuper et al., 2021).

In a global mass-spectrometry-based proteomics screening for m6A readers, human G3BPs (G3BP1 and G3BP2) were identified as proteins that are strongly repelled by N6-methyladenosine (m6A) modification (Edupuganti et al., 2017; Zhao et al., 2017). m6A plays a major role in eukaryotic mRNA fate affecting diverse aspects of RNA metabolism including stability, microRNA biogenesis, splicing and translation (Wang et al., 2014; Zhao et al., 2014; Alarcón et al., 2015; Wang et al., 2015). G3BP1 binding to mRNA is positively associated with mRNA stability and this can be affected by the level of m6A. Therefore, in humans, G3BP1 controls the stability of mRNA in numerous ways. In resting cells, G3BP1 binds to newly transcribed mRNA in the nucleus preventing methylation by competing with the m6A methylation machinery. G3BP1 can also bind to its mRNA targets in the cytoplasm forming ribonucleoprotein granules (RNPs) that are assembled into SGs thereby preventing RNA degradation. Moreover, under certain circumstances, cells can demethylate mRNA, thereby allowing G3BP1 to bind to GGACU-containing mRNA sequences leading to increased stability of those mRNA molecules (Edupuganti et al., 2017).

### G3BPs in Plants

There is sparse literature concerning plant stress granules and the role of G3BPs in the formation of SGs unlike in mammals and Drosophila. The genome of Arabidopsis (TAIR10), encodes eight G3BP members, which are characterized by the presence of conserved NTF2-like and RRM domains (Abulfaraj et al., 2018). Plant G3BPs are likely to be involved in a variety of cellular processes where they co-ordinate signal transduction and post-transcriptional gene regulation and play a key role in the formation of SGs. Several ATG3BPs were shown to be localized to SGs upon heat stress (Reuper et al., 2021). Moreover, a member of ATG3BPs ATG3BP7 (AT5G43960) was found to be localized into SGs upon stress conditions (heat or chemical treatments). Expression of human G3BP in plants co-localizes with TZF1, a plant SG marker protein, indicating that SG formation is conserved among eukaryotes (Krapp et al., 2017). A detailed sub-cellular localization of seven out of the eight members of the G3BP family was carried by transient expression in *Nicotiana benthamiana* leaves. All members localized to the cytoplasm under normal conditions, but ATG3BP4 (AT2G03640) localized also to the nucleus. In addition, ATG3BP6 (AT3G25150), ATG3BP2 (AT1G13730), and ATG3BP1 (AT5G48650) formed granule-like structures under normal conditions, whereas all ATG3BPs formed granule-like structures after heat shock treatment (Reuper et al., 2021).

A detailed protein interaction profiling of seven members of ATG3BP family was studied in the context of the formation of homo- and heterooligomers *in vivo*. Generally, all ATG3BPs members appear to interact with each other under ambient conditions as well as under heat stress condition (Reuper et al., 2021).

**FIGURE 2:**
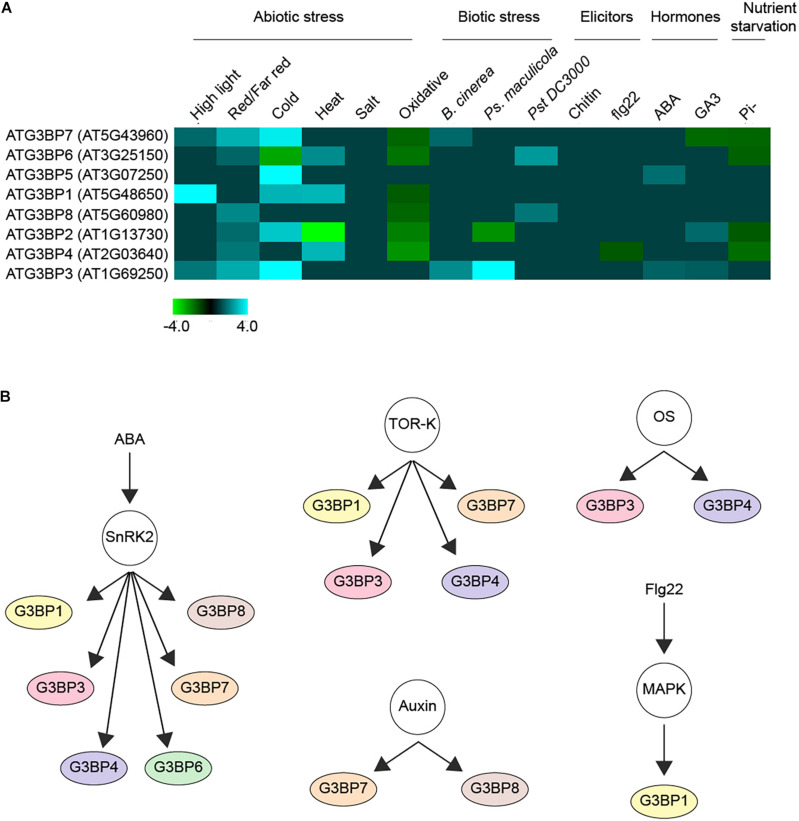
Regulation of G3BPs. **(A)** a heatmap showing the induction or repression (log2 Fold change > ± 1) of the different G3BPs by various abiotic or biotic stresses, elicitors (such as chitin, flg22), hormones (such as ABA, GA3) and nutrient starvation. **(B)** G3BPs are reported as phospho-proteins in several large scale phosphoproteomics studies. The networks show the phosphorylation of different G3BPs under different condition. ABA treatment activates the kinase SnRK2, which inturn phosphorylates several substrates including G3BPs. The TOR-kinase also phosphorylates some G3BPs, so does osmotic stress (OS), auxin treatment or flg22 treatment. All this analysis was carried out using the PhosPhAT database and summarized.

Upon screening around 931 RNAseq studies of Arabidopsis included in Genevestigator (Zimmermann et al., 2004), we observed that the expression profiles of the different members of ATG3BPs differ according to developmental stages and tissue specificity (Abulfaraj et al., 2018). We now extended the screening to 2702 RNAseq studies to look at the expression pattern of the G3BPs in response to diverse perturbations. A summary of gene expression responses to a wide variety of perturbations including abiotic stresses, biotic stresses, elicitors, hormones, and nutrient starvation is represented as a heatmap generated with fold change expression values (log2 Fold change > ± 1) compared to control (Figure 2A). Overall, all the abiotic stresses induce the gene expression of G3BPs except oxidative stress which suppresses the gene expression. ATG3BP 1, 2, 3, 5, and 7 are induced by cold stress. ATG3BP 4 and 7 are induced by high light and red/far red. ATG3BP1 is induced by high light as well as cold and heat stresses. While heat stress induces the expression of ATG3BP 4 and 6, it suppresses the expression of ATG3BP2. Necrotrophic fungus *B. cinereal* induces the expression of ATG3BP3 and 7. While *Pseudomonas syringae maculicola (Psm)* induced the expression of ATG3BP3, it suppresses the expression of ATG3BP2. Hemibiotroph *Pseudomonas syringae* pv. *tomato DC3000* induces the expression ATG3BP6 and 8. Flg22 induces the expression of ATG3BP4. Plant hormone abscisic acid (ABA) induces the expression of ATG3BP 3 and 5. Gibberellic acid GA3 induces the expression of ATG3BP2 and 3 while it suppresses the expression of ATG3BP7. Phosphate starvation suppresses the expression of ATG3BP 2, 6, 4, and 7. Intriguingly, salt and chitin do not affect the gene expression of any of the G3BPs. This shows that members of AtG3BPs have potentially different roles in various stress signaling pathways (Figure 1).

In mammals, G3BPs bind to 40S ribosomes through their RGG region, which is crucial for stress granule condensation. This condensation process is strongly controlled by human Caprin1/USP10 (Kedersha et al., 2016). In bimolecular fluorescence complementation (BiFC) experiments *in planta*, seven out of eight G3BPs showed interaction with AtUBP-24 (AT4G30890), the putative plant homolog of the mammalian USP10 that inhibits SG formation upon binding to G3BP. The Arabidopsis ATG3BPs also form homo- and heterodimerization as reported for human G3BPs (Vognsen et al., 2013; Reuper et al., 2021).

So far only two of the family members have been functionally characterized in plants. While one of them, ATG3BP7 (AT5G43960) was involved in viral immunity (Krapp et al., 2017), the other member, ATG3BP1 (AT5G48650) was shown to be a negative regulator against a bacterial pathogen (Abulfaraj et al., 2018).

## G3BPs and Post-Transcriptional Regulation of mRNA

Organisms respond to environmental stimuli through the activation of signal transduction pathways ultimately altering gene expression and both primary and secondary metabolism. However, besides these mechanisms, recent research revealed important roles of RNA metabolism also contribute to stress adaptation. These processes include alternative splicing, processing, nucleocytoplasmic shuttling, RNA stability and decay and the control of translation, in plant stress biology (Nakaminami et al., 2012; Wang and Chekanova, 2016; Figure 1). Prior to translation, posttranscriptional regulation (PTR) of mRNA serves as a rapid response to cellular stress (Bovaird et al., 2018; Tutucci et al., 2018). The repression of translation during stress activates several post-transcriptional regulatory proteins and mRNAs that induce the formation of condensed membrane-less cytoplasmic organelles defined as granules or foci by phase separation (Protter and Parker, 2016; Van Treeck et al., 2018). mRNAs that are not being translated can aggregate into two types of distinct cytoplasmic stress-induced mRNP granules described as processing bodies (PBs) and stress granules (SGs) (Figure 1). In eukaryotic cells, over 20 different proteins have been discovered in the PBs and more than 70 in the SGs. Moreover, several proteins are common between different RNA granule types both in yeast and mammals (Poblete-Durán et al., 2016). Although it is clear that PBs and SGs in plant, yeast and animal have related functions (Xu and Chua, 2009; Sorenson and Bailey-Serres, 2014), the formation and composition of RNA granules in plants is less clear. A dynamic process, referred to as mRNA cycling, involves mRNPs that can move between polysomes, P-bodies and stress granules for storage and remodeling. Translationally inactive mRNAs were found to accumulate in P-bodies along with the mRNA degradation and translation repression machinery, or in SGs that contain mRNAs stalled in translation initiation (Balagopal and Parker, 2009; Decker and Parker, 2012; Guzikowski et al., 2019).

Active endogenous mRNAs containing the 5′ cap and the 3′ poly(A) tail are generally stable and ready for translation. Each individual transcript has its specific turnover rate, ranging from translation to degradation. Several protein factors within the messenger ribonucleoprotein (mRNP)-complexes accumulate around the mRNAs controlling the fate for each transcript throughout their dynamic life cycle (Mitchell and Parker, 2014). Shortening of the 3′ poly(A) tail by deadenylases and removal of the 5′ cap by decapping protein 2 (DCP2) activates 5′–3′ mRNA decay through exoribonucleases and 3′–5′ degradation through the exosome that harbors both exo- and endoribonuclease activities. The interaction and aggregation of mRNA binding proteins allows the formation of RNA granules. Deadenylation, decapping, and degradation activities coalesce in RNA granules. Several proteins identified as endogenous RNA silencing suppressors were found to be involved in 5′–3′ and 3′–5′ RNA degradation (Mäkinen et al., 2017). There is often competition between mRNA translation and degradation correlating with the location of the mRNPs depending on whether the mRNPs are engaged in translation or assembled with the decapping machinery. P-bodies are cytoplasmic RNA-protein granules which are formed as a result of the accumulation of non-translating mRNAs, which include elements that recruit P-body components. Although the complete composition of P-bodies has not been well-studied, P-bodies contain translation repressors, mRNA degradation enzymes, and cofactors including decapping enzymes, activators of decapping complex, and exoribonucleases. In addition, P-bodies also contain mRNAs and proteins responsible for Nonsense mediated decay (NMD) (Guzikowski et al., 2019).

SGs are large evolutionarily conserved cytoplasmic aggregates of proteins and untranslated mRNAs formed in response to stresses such as viral infections, heat, oxidation, and starvation as a result of translational repression. Formation of SGs results from the activation of one of the eIF2 kinases by oxidative, heat stress or nutrient deficiency leading to the phosphorylation of the alpha subunit of eIF2 and thereby blocking translation by accumulating initiation complexes around the transcripts (Krapp et al., 2017). Animal, plant, and yeast SGs contain translation initiation components and play an important role in modulating the stress translatome and proteome by selective storage of mRNAs and protection of proteins. The accumulation of translationally inactive mRNAs in SGs inhibits translation and subsequently their protein activity. Moreover, SGs can disassemble and allow rapid reactivation and release of mRNAs into a translationally active form upon stress recovery (Parker, 2012; Merret et al., 2013; Protter and Parker, 2016; Kosmacz et al., 2019). Since several SG residents in mammals and yeast include ATP−dependent helicases and remodelers, formation of SGs is probably an ATP−driven process that involves RNA remodeling (Courchaine et al., 2016; Banani et al., 2017).

Although it has been widely accepted that most non-translating RNAs are diverted to SGs, a recent study challenges this long-standing knowledge by providing evidence that SG-localized transcripts can undergo translation and there is continuous translocation of mRNAs between cytosol and SGs (Mateju et al., 2020).

## Plant Stress Granules

Although much advance has been made recently to identify the composition of plant SGs and the physical characteristics of their formation, little is known on the functional machinery and how significantly SGs contribute to plant stress adaptation. Most of the Arabidopsis SG components have been identified by their homology with animal and yeast SGs (Chantarachot and Bailey-Serres, 2018). Several proteomic analyses identified Arabidopsis SG resident proteins, some of which are important players in the assembly and dynamics of SGs such as proteins involved in in RNA−binding [TUDOR−SN proteins (TSN1 and TSN2)] (Gutierrez-Beltran et al., 2015; Kosmacz et al., 2019), UBP1, poly(A)−binding proteins 2 and 8 (PAB2 and PAB8), proteins possessing prion−like (PrLD) and ATPase regions, elongation initiation factors (eIFs), chaperones TCP−1 complex, heat shock proteins, RNA/DNA helicases, and a variety of RNA−binding proteins (Gilks et al., 2004; Weber et al., 2008; Pomeranz et al., 2010; Sorenson and Bailey-Serres, 2014; Jain et al., 2016; Protter and Parker, 2016; Kosmacz et al., 2019; Tabassum et al., 2020). Additionally, key enzymes responsible for several pathways including ethylene, glucosinolate, and rhamnose metabolism were also identified as components of SGs (Kosmacz et al., 2019). Furthermore, signaling proteins involved in plant growth e.g., cyclin−dependent kinase 1 [CDKA1] and stress response proteins such as mitogen-activated protein kinases and glutathione *S*−transferases were also found to be residents of SGs (Kosmacz et al., 2019).

## G3BPs in Plant Viral Infections

Several studies demonstrated that plant viruses require stress granules and processing bodies for effective replication and translation. The plant antiviral RNA silencing machinery is important for the regulation of viral RNA stability and expression. This in turn is suppressed by viral RNA silencing machinery (Shukla et al., 2019). However, the plant cellular mRNA regulatory network utilized by viruses remains unclear. Viruses were reported to suppress the formation of SGs for replication purposes. SGs are antiviral compartments and many viruses were reported to suppress the formation of SGs for replication purpose through G3BP interactions. Multifunctional G3BPs contribute to the formation of SG upstream of eIF2α phosphorylation (Mäkinen et al., 2017). A role of SGs and G3BPs has been found in a number of viral studies in animals and recently also in plants. Viruses target G3BPs to block the formation of SGs. Proteins that contain the short linear motif Phe-Gly-Asp-Phe (FGDF) bind G3BPs via the hydrophobic groove on the surface of the NTF2-like domain thereby blocking the ability of G3BPs to form SGs and facilitating the replication of viruses harboring this motif (McInerney, 2015). Although FGDF-like binding motifs have been found in some plant viruses, the interaction of plant G3BPs with viruses is poorly investigated (Panas et al., 2015). Since SGs are normally coupled with the regulation of gene expression, viruses have evolved different mechanisms to neutralize their assembly and take advantages of them for effective replication. ATG3BP7 (AT5G43960) was identified as an Arabidopsis protein homolog to HsG3BP and is a stress granule component that plays a role in plant virus resistance. ATG3BP7 was recognized throughout its interaction with a viral protein, the nuclear shuttle protein 3 (NSP3) of the abutilon mosaic virus in SGs (Krapp et al., 2017; Reuper et al., 2021). Interaction between ATG3BP7 and viral proteins was found to be through a conserved FGDF-type motif. FGDF motifs are found in the proteases of potyviruses, waikaviruses, and closteroviruses proposing that formation of SG through G3BP interactions could help in understanding the interactions in plant virus infections (Mäkinen et al., 2017). ATG3BP7 fused to GFP co-localized with TZFI, a plant SG marker protein. Transient expression of ATG3BP7 fused to GFP induces the formation of SGs upon stress (heat shock or chemical treatment). ATG3BP7 also has an RGG motif in its C-terminal similar to HsG3BP, which interacts with 40S ribosomal subunits, mediating SG formation. Moreover, as shown in mammals, several plant viral proteins have the ability to bind to ATG3BP7, preventing the formation of SGs. For example, ATG3BP7 colocalizes with the nuclear shuttle protein (NSP) of the begomovirus Abutilon mosaic virus (AbMV), which has an “FVSF” motif at its C-terminal end, and the NSP of the nanovirus pea necrotic yellow dwarf virus with the “FNGSF” motif, in plant cells, respectively, upon stress (Krapp et al., 2017; Reuper et al., 2021). P1 protease of Turnip mosaic virus (P1-TuMV) contains an FGSF-motif and FGSL-motif at its N-terminus. This motif known as a binding site for G3BP leading to the formation of SG and usually targeted by viruses to suppress the formation of SG. G3BP7 were co localized with the P1 of two TuMV isolates, which are United Kingdom 1 and DEU 2. P1-TuMV-DEU 2 was co-localized with ATG3BP-7 under abiotic stress conditions, whereas P1-TuMV-UK 1 did not (Reuper and Krenz, 2021). Furthermore, ATG3BPs behave similarly to their mammalian counterparts in being expressed early upon virus infection. It has been also reported that helper proteinase (HC-pro) of potato virus A (PVA) is able to induce the formation of RNA granules (Hafrén et al., 2015).

## G3BPs in Plant Stomatal Immunity

ATG3BP1 (AT5G48650) is an Arabidopsis protein that plays a role in plant immunity. Arabidopsis loss of function mutants in this gene showed enhanced resistance to the virulent bacterial pathogen *Pseudomonas syringae* pv. *tomato*. Pathogen resistance is mediated in ATG3BP1 mutants by altered stomatal and apoplastic immunity, restricting pathogen entry into stomates and showing insensitivity to bacterial coronatine–mediated stomatal reopening. Moreover, ATG3BP1 mutants accumulate H_2_O_2_ and show constitutive upregulation of a number of defense marker genes including *PR1*, indicating that ATG3BP1 functions as a negative regulator of immunity. The resistance phenotype of ATG3BP1 mutant plants depends on the up-regulation of salicylic acid biosynthesis and signaling but does not come with a growth penalty, which makes ATG3BP1 a highly interesting target for molecular breeding of pathogen-resistant crops (Abulfaraj et al., 2018).

## Regulation of Plant G3BPs

The adaptation of plants to environmental stresses involves complex signaling networks that induce cellular, physiological and developmental responses (Ramirez-Prado et al., 2018). Several types of post-translational protein modifications have been studied including acetylation, thiolation, adenylation, ribosylation and phosphorylation. PTMs regulate signal transduction pathways in all biological processes including metabolism, growth, division, differentiation, motility, stability, subcellular localization, and immunity (Rayapuram et al., 2021). Upon activation, G3BPs can play a multitude of roles such as exerting transcriptional control by modulating the expression of key stress genes (Abulfaraj et al., 2018; Figure 1).

Protein kinases including mitogen-activated protein kinases (MAPKs), extracellular signal-regulated kinases (ERK), and cyclin-dependent kinases (CDKs) function in transferring γ-phosphate from ATP to specific amino acids in proteins. 30% of all cellular proteins are phosphorylated on at least one serine, threonine or tyrosine (S/T/Y) residue in the phosphorylation site (Ubersax and Ferrell, 2007; Ghelis, 2011; Lata et al., 2015). A thorough investigation of the PhosPhAT database revealed that seven out of the eight members of the plant G3BPs were found to be phosphorylated in several large scale phosphoproteomic analysis as shown in Figure 2B (Heazlewood et al., 2007). This suggests that the G3BPs are most probably regulated via phosphorylation as a post-translational modification. They were found to be phosphorylated in response to several process including osmotic stress, RNA metabolism, seed maturation, microtubule stability and plant growth. Others were found to respond to plant hormones such as auxin, abscisic acid, and ethylene. In addition, some of them were found to be substrates of several protein kinases including SnRK2 protein kinase and mitogen-Activated Protein Kinases (MAPKs) (Heazlewood et al., 2007).

G3BPs can also induce alternative splicing by binding to their cognate mRNAs via the RGG (Arginine-glycine rich) domain that is often found in RNA-binding proteins (Darnell et al., 2001). G3BPs play a vital role in the formation and maintenance of SGs owing to their ability to bind to RNA. Mutants lacking G3BPs lose the ability to form SGs. Once disassembled, SGs could then release mRNAs into a translationally active form leading to protein synthesis (Kedersha and Anderson, 2002; Guillén-Boixet et al., 2020). However, it is still not clear how distinct species of mRNAs are regulated at the translational level and whether this specificity is conferred by G3BPs and further work is needed to understand the functions of distinct G3BPs in plants.

## Conclusion

The field of plant G3BPs research is still in its infancy. So far, not much has been known about them nor have any of them been characterized in detail to understand their molecular function. However, since G3BPs form a protein family that is highly conserved during evolution and given the diverse roles G3BPs play in yeast and mammals, it will be interesting to see what functions have been conserved or evolved during the expansion of G3BPs in plants. Research in the mammalian field has proven beyond doubt that G3BPs bind to their specific mRNA targets in the nucleus and influence their fate post-transcriptionally in response to various environmental and cellular signals. It will be interesting to find out if the significantly higher number of G3BPs in plants bring about specificity with respect to the RNAs that they bind to or by responding to different upstream environmental and cellular cues. We still do not understand the mechanisms that contribute to the recognition of mRNAs by the G3BPs. There is also unequivocal evidence to prove that plant G3BPs localize to SGs as in mammals, but there is scant evidence on the precise composition or the function of SGs in plants. Characterization of plants G3BPs in the formation of SGs will lead to a better understanding of how plants respond to stress. So far, SGs were considered to be storage centers for mRNAs to vade through unfavorable conditions but recent findings challenge this very simplistic notion. Further research is also warranted into the roles of G3BPs and SGs in the interaction between plants and viruses. A major unanswered question is, how are the G3BPs regulated? Evidence suggests that they are regulated both transcriptionally as well as post-translationally. These investigations will further our understanding of how translational control mechanisms contribute to the global regulation of gene expression in plant stress responses.

## Author Contributions

AA, HH, and NR wrote the article. AA, HH, and NR revised the work. All authors contributed to this work.

## Conflict of Interest

The authors declare that the research was conducted in the absence of any commercial or financial relationships that could be construed as a potential conflict of interest.

## References

[B1] Abulfaraj A. A. Mariappan K. Bigeard J. Manickam P. Blilou I. Guo X. (2018). The *Arabidopsis* homolog of human G3BP1 is a key regulator of stomatal and apoplastic immunity. *Life Sci. Allian.* 1:e201800046. 10.26508/lsa.201800046 30456348 PMC6238584

[B2] Alam U. Kennedy D. (2019). Rasputin a decade on and more promiscuous than ever? A review of G3BPs. *Biochim. Biophys. Acta* 1866 360–370. 10.1016/j.bbamcr.2018.09.001 30595162 PMC7114234

[B3] Alarcón C. R. Lee H. Goodarzi H. Halberg N. Tavazoie S. F. (2015). N 6-methyladenosine marks primary microRNAs for processing. *Nature* 519 482–485. 10.1038/nature14281 25799998 PMC4475635

[B4] Anisimov S. Takahashi M. Kakihana T. Katsuragi Y. Kitaura H. Zhang L. (2019). G3BP1 inhibits ubiquitinated protein aggregations induced by p62 and USP10. *Sci. Rep.* 9:12896. 10.1038/s41598-019-46237-1 PMC6733845 31501480

[B5] Balagopal V. Parker R. (2009). Polysomes, P bodies and stress granules: states and fates of eukaryotic mRNAs. *Curr. Opin. Cell Biol.* 21 403–408. 10.1016/j.ceb.2009.03.005 19394210 PMC2740377

[B6] Banani S. F. Lee H. O. Hyman A. A. Rosen M. K. (2017). Biomolecular condensates: organizers of cellular biochemistry. *Nat. Rev. Mol. Cell Biol.* 18 285–298. 10.1038/nrm.2017.7 28225081 PMC7434221

[B7] Bovaird S. Patel D. Padilla J. C. A. Lécuyer E. (2018). Biological functions, regulatory mechanisms, and disease relevance of RNA localization pathways. *FEBS Lett.* 592 2948–2972. 10.1002/1873-3468.13228 30132838

[B8] Chantarachot T. Bailey-Serres J. (2018). Polysomes, stress granules, and processing bodies: a dynamic triumvirate controlling cytoplasmic mRNA fate and function. *Plant Physiol.* 176 254–269. 10.1104/pp.17.01468 29158329 PMC5761823

[B9] Cléry A. Blatter M. Allain F. H. T. (2008). RNA recognition motifs: boring? Not quite. *Curr. Opin. Struct. Biol.* 18 290–298. 10.1016/j.sbi.2008.04.002 18515081

[B10] Courchaine E. M. Lu A. Neugebauer K. M. (2016). Droplet organelles? *EMBO J.* 35 1603–1612. 10.15252/embj.201593517 27357569 PMC4969579

[B11] Darnell J. C. Jensen K. B. Jin P. Brown V. Warren S. T. Darnell R. B. (2001). Fragile X mental retardation protein targets G quartet mRNAs important for neuronal function. *Cell* 107 489–499. 10.1016/s0092-8674(01)00566-9 11719189

[B12] Decker C. J. Parker R. (2012). P-bodies and stress granules: possible roles in the control of translation and mRNA degradation. *Cold Spring Harb. Perspect. Biol.* 4:a012286. 10.1101/cshperspect.a012286 22763747 PMC3428773

[B13] Edupuganti R. R. Geiger S. Lindeboom R. G. H. Shi H. Hsu P. J. Lu Z. (2017). N 6-methyladenosine (m 6 A) recruits and repels proteins to regulate mRNA homeostasis. *Nat. Struct. Mol. Biol.* 24 870–878. 10.1038/nsmb.3462 28869609 PMC5725193

[B14] Fros J. J. Geertsema C. Zouache K. Baggen J. Domeradzka N. Van Leeuwen D. M. (2015). Mosquito Rasputin interacts with chikungunya virus nsP3 and determines the infection rate in *Aedes albopictus*. *Parasit. Vect.* 8:464. 10.1186/s13071-015-1070-4 PMC4573678 26384002

[B15] Ghelis T. (2011). Signal processing by protein tyrosine phosphorylation in plants. *Plant Signal. Behav.* 6 942–951. 10.4161/psb.6.7.15261 21628997 PMC3257767

[B16] Gilks N. Kedersha N. Ayodele M. Shen L. Stoecklin G. Dember L. M. (2004). Stress granule assembly is mediated by prion-like aggregation of TIA-1. *Mol. Biol. Cell* 15 5383–5398. 10.1091/mbc.e04-08-0715 15371533 PMC532018

[B17] Götte B. Panas M. D. Hellström K. Liu L. Samreen B. Larsson O. (2019). Separate domains of G3BP promote efficient clustering of alphavirus replication complexes and recruitment of the translation initiation machinery. *PLoS Pathog.* 15:e1007842. 10.1371/journal.ppat.1007842 31199850 PMC6594655

[B18] Götte B. Utt A. Fragkoudis R. Merits A. Mcinerney G. M. (2020). Sensitivity of alphaviruses to G3BP deletion correlates with efficiency of replicase polyprotein processing. *J. Virol.* 94 e01681-19. 10.1128/JVI.01681-19 PMC7081891 31941782

[B19] Guillén-Boixet J. Kopach A. Holehouse A. S. Wittmann S. Jahnel M. Schlüßler R. (2020). RNA-induced conformational switching and clustering of G3BP drive stress granule assembly by condensation. *Cell* 181 346–361. 10.1016/j.cell.2020.03.049 32302572 PMC7181197

[B20] Gutierrez-Beltran E. Moschou P. N. Smertenko A. P. Bozhkov P. V. (2015). Tudor staphylococcal nuclease links formation of stress granules and processing bodies with mRNA catabolism in *Arabidopsis*. *Plant Cell* 27 926–943. 10.1105/tpc.114.134494 25736060 PMC4558657

[B21] Guzikowski A. R. Chen Y. S. Zid B. M. (2019). Stress-induced mRNP granules: form and function of processing bodies and stress granules. *Wiley Interdis. Rev. RNA* 10:e1524. 10.1002/wrna.1524 30793528 PMC6500494

[B22] Hafrén A. Lõhmus A. Mäkinen K. (2015). Formation of Potato virus A-induced RNA granules and viral translation are interrelated processes required for optimal virus accumulation. *PLoS Pathog.* 11:e1005314. 10.1371/journal.ppat.1005314 26641460 PMC4671561

[B23] Heazlewood J. L. Durek P. Hummel J. Selbig J. Weckwerth W. Walther D. (2007). PhosPhAt: a database of phosphorylation sites in *Arabidopsis thaliana* and a plant-specific phosphorylation site predictor. *Nucleic Acids Res.* 36 D1015–D1021. 17984086 10.1093/nar/gkm812 PMC2238998

[B24] Irvine K. Stirling R. Hume D. Kennedy D. (2004). Rasputin, more promiscuous than ever: a review of G3BP. *Int. J. Dev. Biol.* 48 1065–1077. 10.1387/ijdb.041893ki 15602692

[B25] Jain S. Wheeler J. R. Walters R. W. Agrawal A. Barsic A. Parker R. (2016). ATPase-modulated stress granules contain a diverse proteome and substructure. *Cell* 164 487–498. 10.1016/j.cell.2015.12.038 26777405 PMC4733397

[B26] Kedersha N. Anderson P. (2002). Stress granules: sites of mRNA triage that regulate mRNA stability and translatability. *Biochem. Soc. Trans.* 30 963–969. 10.1042/bst0300963 12440955

[B27] Kedersha N. Panas M. D. Achorn C. A. Lyons S. Tisdale S. Hickman T. (2016). G3BP–Caprin1–USP10 complexes mediate stress granule condensation and associate with 40S subunits. *J. Cell Biol.* 212 845–860. 27022092 10.1083/jcb.201508028 PMC4810302

[B28] Kennedy D. French J. Guitard E. Ru K. Tocque B. Mattick J. (2002). Characterization of G3BPs: tissue specific expression, chromosomal localisation and rasGAP120 binding studies. *J. Cell. Biochem.* 84 173–187. 10.1002/jcb.1277 11746526

[B29] Kim D. Y. Reynaud J. M. Rasalouskaya A. Akhrymuk I. Mobley J. A. Frolov I. (2016). New world and old world alphaviruses have evolved to exploit different components of stress granules, FXR and G3BP proteins, for assembly of viral replication complexes. *PLoS Pathog.* 12:e1005810. 10.1371/journal.ppat.1005810 27509095 PMC4980055

[B30] Kosmacz M. Gorka M. Schmidt S. Luzarowski M. Moreno J. C. Szlachetko J. (2019). Protein and metabolite composition of *Arabidopsis* stress granules. *New Phytol.* 222 1420–1433. 10.1111/nph.15690 30664249

[B31] Krapp S. Greiner E. Amin B. Sonnewald U. Krenz B. (2017). The stress granule component G3BP is a novel interaction partner for the nuclear shuttle proteins of the nanovirus pea necrotic yellow dwarf virus and geminivirus abutilon mosaic virus. *Virus Res.* 227 6–14. 10.1016/j.virusres.2016.09.021 27693920

[B32] Lata C. Muthamilarasan M. Prasad M. (2015). “Drought stress responses and signal transduction in plants,” in *Elucidation of Abiotic Stress Signaling in Plants*, ed. Pandey G. K. (Cham: Springer), 195–225. 10.1007/978-1-4939-2540-7_7

[B33] Laver J. D. Ly J. Winn A. K. Karaiskakis A. Lin S. Nie K. (2020). The RNA-binding protein Rasputin/G3BP enhances the stability and translation of its target mRNAs. *Cell Rep.* 30 3353–3367. 10.1016/j.celrep.2020.02.066 32160542

[B34] Mäkinen K. Lõhmus A. Pollari M. (2017). Plant RNA regulatory network and RNA granules in virus infection. *Front. Plant Sci.* 8:2093. 10.3389/fpls.2017.02093 29312371 PMC5732267

[B35] Martin S. Zekri L. Metz A. Maurice T. Chebli K. Vignes M. (2013). Deficiency of G3BP1, the stress granules assembly factor, results in abnormal synaptic plasticity and calcium homeostasis in neurons. *J. Neurochem.* 125 175–184. 10.1111/jnc.12189 23373770

[B36] Mateju D. Eichenberger B. Voigt F. Eglinger J. Roth G. Chao J. A. (2020). Single-molecule imaging reveals translation of mRNAs localized to stress granules. *Cell* 183 1801–1812. 10.1016/j.cell.2020.11.010 33308477

[B37] McInerney G. M. (2015). FGDF motif regulation of stress granule formation. *DNA Cell Biol.* 34 557–560. 10.1089/dna.2015.2957 26101899

[B38] Merret R. Descombin J. Juan Y.-T. Favory J.-J. Carpentier M.-C. Chaparro C. (2013). XRN4 and LARP1 are required for a heat-triggered mRNA decay pathway involved in plant acclimation and survival during thermal stress. *Cell Rep.* 5 1279–1293. 10.1016/j.celrep.2013.11.019 24332370

[B39] Mitchell S. F. Parker R. (2014). Principles and properties of eukaryotic mRNPs. *Mol. Cell* 54 547–558. 10.1016/j.molcel.2014.04.033 24856220

[B40] Nakaminami K. Matsui A. Shinozaki K. Seki M. (2012). RNA regulation in plant abiotic stress responses. *Biochim. Biophys. Acta* 1819 149–153. 21840431 10.1016/j.bbagrm.2011.07.015

[B41] Nichols R. C. Wang X. W. Tang J. Hamilton B. J. High F. A. Herschman H. R. (2000). The RGG domain in hnRNP A2 affects subcellular localization. *Exp. Cell Res.* 256 522–532. 10.1006/excr.2000.4827 10772824

[B42] Pamonsinlapatham P. Hadj-Slimane R. Lepelletier Y. Allain B. Toccafondi M. Garbay C. (2009). p120-Ras GTPase activating protein (RasGAP): a multi-interacting protein in downstream signaling. *Biochimie* 91 320–328. 10.1016/j.biochi.2008.10.010 19022332

[B43] Panas M. D. Schulte T. Thaa B. Sandalova T. Kedersha N. Achour A. (2015). Viral and cellular proteins containing FGDF motifs bind G3BP to block stress granule formation. *PLoS Pathog.* 11:e1004659. 10.1371/journal.ppat.1004659 25658430 PMC4450067

[B44] Parker F. Maurier F. Delumeau I. Duchesne M. Faucher D. Debussche L. (1996). A Ras-GTPase-activating protein SH3-domain-binding protein. *Mol. Cell Biol.* 16 2561–2569. 8649363 10.1128/mcb.16.6.2561 PMC231246

[B45] Parker R. (2012). RNA degradation in *Saccharomyces cerevisae*. *Genetics* 191 671–702. 10.1534/genetics.111.137265 22785621 PMC3389967

[B46] Poblete-Durán N. Prades-Pérez Y. Vera-Otarola J. Soto-Rifo R. Valiente-Echeverría F. (2016). Who regulates whom? An overview of RNA granules and viral infections. *Viruses* 8:180. 10.3390/v8070180 27367717 PMC4974515

[B47] Pomeranz M. Lin P.-C. Finer J. Jang J.-C. (2010). AtTZF gene family localizes to cytoplasmic foci. *Plant Signal. Behav.* 5 190–192. 10.4161/psb.5.2.10988 20173417 PMC2884132

[B48] Protter D. S. W. Parker R. (2016). Principles and properties of stress granules. *Trends Cell Biol.* 26 668–679. 10.1016/j.tcb.2016.05.004 27289443 PMC4993645

[B49] Quimby B. B. Lamitina T. L’hernault S. W. Corbett A. H. (2000). The mechanism of ran import into the nucleus by nuclear transport factor 2. *J. Biol. Chem.* 275 28575–28582. 10.1074/jbc.m005055200 10889207

[B50] Ramirez-Prado J. S. Abulfaraj A. A. Rayapuram N. Benhamed M. Hirt H. (2018). Plant immunity: from signaling to epigenetic control of defense. *Trends Plant Sci.* 23 833–844. 10.1016/j.tplants.2018.06.004 29970339

[B51] Rayapuram N. Jarad M. Alhoraibi H. M. Bigeard J. Abulfaraj A. A. Völz R. (2021). Chromatin phosphoproteomics unravels a function for AT-hook motif nuclear localized protein AHL13 in PAMP-triggered immunity. *Proc. Natl. Acad. Sci.* 118:e2004670118. 10.1073/pnas.2004670118 33419940 PMC7826357

[B52] Ren R. Mayer B. J. Cicchetti P. Baltimore D. (1993). Identification of a ten-amino acid proline-rich SH3 binding site. *Science* 259 1157–1161. 10.1126/science.8438166 8438166

[B53] Reuper H. Amari K. Krenz B. (2021). Analyzing the G3BP-like gene family of *Arabidopsis thaliana* in early turnip mosaic virus infection. *Sci. Rep.* 11:2187. 10.1038/s41598-021-81276-7 PMC7838295 33500425

[B54] Reuper H. Krenz B. (2021). Comparison of two *Turnip mosaic virus* P1 proteins in their ability to co-localize with the *Arabidopsis thaliana* G3BP-2 protein. *Virus Genes* 57 233–237. 10.1007/s11262-021-01829-w 33599903 PMC7985126

[B55] Scholte F. E. M. Tas A. Albulescu I. C. Žusinaite E. Merits A. Snijder E. J. (2015). Stress granule components G3BP1 and G3BP2 play a proviral role early in Chikungunya virus replication. *J. Virol.* 89 4457–4469. 10.1128/jvi.03612-14 25653451 PMC4442398

[B56] Schulte T. Liu L. Panas M. D. Thaa B. Dickson N. Götte B. (2016). Combined structural, biochemical and cellular evidence demonstrates that both FGDF motifs in alphavirus nsP3 are required for efficient replication. *Open Biol.* 6 160078. 10.1098/rsob.160078 27383630 PMC4967826

[B57] Shukla A. López-González S. Hoffmann G. Hafrén A. (2019). Diverse plant viruses: a toolbox for dissection of cellular pathways. *J. Exp. Bot.* 70 3029–3034. 10.1093/jxb/erz122 30882863 PMC6598076

[B58] Sorenson R. Bailey-Serres J. (2014). Selective mRNA sequestration by OLIGOURIDYLATE-BINDING PROTEIN 1 contributes to translational control during hypoxia in *Arabidopsis*. *Proc. Natl. Acad. Sci. U.S.A.* 111 2373–2378. 10.1073/pnas.1314851111 24469793 PMC3926019

[B59] Suyama M. Doerks T. Braun I. C. Sattler M. Izaurralde E. Bork P. (2000). Prediction of structural domains of TAP reveals details of its interaction with p15 and nucleoporins. *EMBO Rep.* 1 53–58. 10.1093/embo-reports/kvd009 11256625 PMC1083685

[B60] Tabassum N. Eschen−Lippold L. Athmer B. Baruah M. Brode M. Maldonado−Bonilla L. D. (2020). Phosphorylation−dependent control of an RNA granule−localized protein that fine−tunes defence gene expression at a post−transcriptional level. *Plant J.* 101 1023–1039. 10.1111/tpj.14573 31628867

[B61] Tourrière H. Chebli K. Zekri L. Courselaud B. Blanchard J. M. Bertrand E. (2003). The RasGAP-associated endoribonuclease G3BP assembles stress granules. *J. Cell Biol.* 160 823–831. 10.1083/jcb.200212128 12642610 PMC2173781

[B62] Tutucci E. Vera M. Singer R. H. (2018). Single-mRNA detection in living *S. cerevisiae* using a re-engineered MS2 system. *Nat. Protoc.* 13 2268–2296. 10.1038/s41596-018-0037-2 30218101

[B63] Ubersax J. A. Ferrell J. E. Jr. (2007). Mechanisms of specificity in protein phosphorylation. *Nat. Rev. Mol. Cell Biol.* 8 530–541. 17585314 10.1038/nrm2203

[B64] Van Treeck B. Protter D. S. W. Matheny T. Khong A. Link C. D. Parker R. (2018). RNA self-assembly contributes to stress granule formation and defining the stress granule transcriptome. *Proc. Natl. Acad. Sci. U.S.A.* 115 2734–2739. 10.1073/pnas.1800038115 29483269 PMC5856561

[B65] Vognsen T. Møller I. R. Kristensen O. (2013). Crystal structures of the human G3BP1 NTF2-like domain visualize FxFG Nup repeat specificity. *PLoS One* 8:e80947. 10.1371/journal.pone.0080947 24324649 PMC3852005

[B66] Wang H. L. V. Chekanova J. A. (2016). Small RNAs: essential regulators of gene expression and defenses against environmental stresses in plants. *Wiley Interdiscip. Rev. RNA* 7 356–381. 10.1002/wrna.1340 26924473 PMC6671677

[B67] Wang X. Lu Z. Gomez A. Hon G. C. Yue Y. Han D. (2014). N 6-methyladenosine-dependent regulation of messenger RNA stability. *Nature* 505 117–120. 10.1038/nature12730 24284625 PMC3877715

[B68] Wang X. Zhao B. S. Roundtree I. A. Lu Z. Han D. Ma H. (2015). N6-methyladenosine modulates messenger RNA translation efficiency. *Cell* 161 1388–1399. 10.1016/j.cell.2015.05.014 26046440 PMC4825696

[B69] Weber C. Nover L. Fauth M. (2008). Plant stress granules and mRNA processing bodies are distinct from heat stress granules. *Plant J.* 56 517–530. 10.1111/j.1365-313x.2008.03623.x 18643965

[B70] Xu J. Chua N.-H. (2009). *Arabidopsis* decapping 5 is required for mRNA decapping, P-body formation, and translational repression during postembryonic development. *Plant Cell* 21 3270–3279. 10.1105/tpc.109.070078 19855049 PMC2782270

[B71] Zhang H. Ma Y. Zhang S. Liu H. He H. Li N. (2015). Involvement of Ras GTPase-activating protein SH3 domain-binding protein 1 in the epithelial-to-mesenchymal transition-induced metastasis of breast cancer cells via the Smad signaling pathway. *Oncotarget* 6:17039. 10.18632/oncotarget.3636 25962958 PMC4627290

[B72] Zhao B. S. Wang X. Beadell A. V. Lu Z. Shi H. Kuuspalu A. (2017). m 6 A-dependent maternal mRNA clearance facilitates zebrafish maternal-to-zygotic transition. *Nature* 542 475–478. 10.1038/nature21355 28192787 PMC5323276

[B73] Zhao X. Yang Y. Sun B.-F. Shi Y. Yang X. Xiao W. (2014). FTO-dependent demethylation of N6-methyladenosine regulates mRNA splicing and is required for adipogenesis. *Cell Res.* 24 1403–1419. 10.1038/cr.2014.151 25412662 PMC4260349

[B74] Zimmermann P. Hirsch-Hoffmann M. Hennig L. Gruissem W. (2004). GENEVESTIGATOR. *Arabidopsis* microarray database and analysis toolbox. *Plant Physiol.* 136 2621–2632. 10.1104/pp.104.046367 15375207 PMC523327

